# Supplementary material for: Individual herpes simplex virus 1 (HSV-1) particles exit by exocytosis and accumulate at preferential egress sites
Source: J Virol. 2024 Jan 9;98(2):e01785-23. doi: 10.1128/jvi.01785-23 (PMC10883806; doi:10.1128/jvi.01785-23)
Supplement: Supplemental Material 1 — Supplemental experimental details and results describing PCR validation of HSV-1 recombinant viruses. [file jvi.01785-23-s0001.docx]

PCR of Recombinant HSV-1 Strains to Detect pHluorin Sequence

All PCR samples were prepped using Taq PCR Master Mix Kit (Qiagen) using 1.0 μg of DNA and one primer pair. All PCR reactions were performed using the following conditions: 94°C for 3 min (initial denaturation); 35 cycles of 94°C for 45 s, 54°C for 45 s, and 72°C for 1 min; followed by a final extension at 72°C for 10 minutes. Primers utilized are listed below (Table 1), in the pairwise fashion noted to produce PCR products (Table 2).

PCR samples were purified for sequencing using QIAquick PCR purification kit (Qiagen), following manufacturer protocol.

**Table 1**: Primer Sequences

| **Label** | **Shorthand** | **5’-3’** |
| --- | --- | --- |
| HSV-gM-pHluorin_V1_forward | Primer 1 | CGGTTTCCCTGCTTTTACGC |
| HSV-pHluorin_reverse | Primer 2 | TGCCGGTGGTGCAGATAAAC |
| HSV-pHluorin_forward | Primer 3 | ACATGGTCCTGCTGGAGTTC |
| HSV-gM-pHluorin_V1_reverse | Primer 4 | TGCAGCAACCAAGAGCAGAC |
| HSV-gM-pHluorin_V2_forward | Primer 5 | CCCAGAGGATCTCCCGACTC |
| HSV-gM-pHluorin_V2_reverse | Primer 6 | TACATAAGTGCCCACAAGGCTC |

**Table 2**: Primer Pairs

| **Primer Pair** | **Label** | **Sequence 5’-3’** |
| --- | --- | --- |
| 1 | HSV-gM-pHluorin_V1_forward | CGGTTTCCCTGCTTTTACGC |
|  | HSV-pHluorin_reverse | TGCCGGTGGTGCAGATAAAC |
| 2 | HSV-pHluorin_forward | ACATGGTCCTGCTGGAGTTC |
|  | HSV-gM-pHluorin_V1_reverse | TGCAGCAACCAAGAGCAGAC |
| 3 | HSV-gM-pHluorin_V2_forward | CCCAGAGGATCTCCCGACTC |
|  | HSV-pHluorin_reverse | TGCCGGTGGTGCAGATAAAC |
| 4 | HSV-pHluorin_forward | ACATGGTCCTGCTGGAGTTC |
|  | HSV-gM-pHluorin_V2_reverse | TACATAAGTGCCCACAAGGCTC |

Results of HSV-1 gM-pHluorin PCR

Correct insertion of pHluorin into the gM locus was determined by the band size of the PCR product. PCR products were separated with agarose gel electrophoresis and then visualized with UV light. The PCR products for HSV-1 IH01, HSV-1 IH02, and the gM-pHluorin shuttle plasmid produced the same banding pattern, indicating that the insert sequence from the gM-pHluorin shuttle plasmid successfully recombined into the HSV-1 genome (A-B).


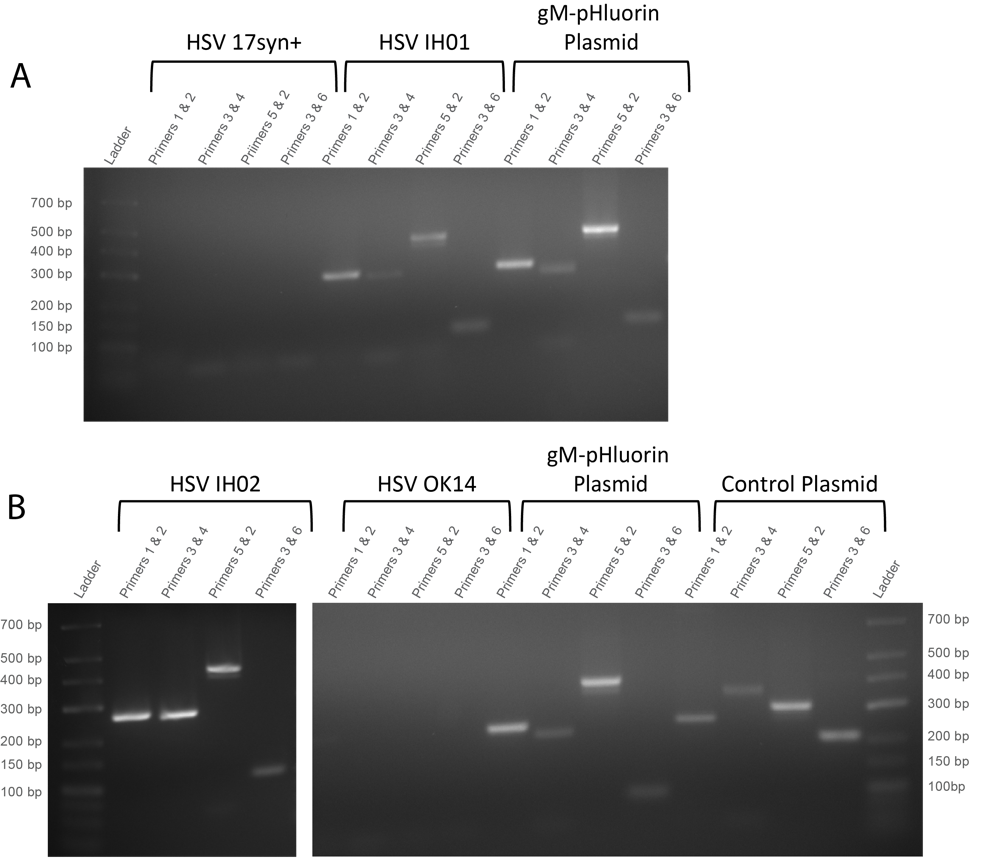


Supplemental Figure 1. PCR products from parental and recombinant HSV-1 strains. A. PCR products of the gM-Phluorin insert sequence with no bands for parental HSV-1 17syn+. Recombinant gM-pHluorin HSV-1 IH01 and the gM-pHluorin plasmid yield two bands at approximately 300 bp, one at ~500bp, and one at ~100 bp. B. PCR of recombinant HSV-1 IH02 with gM-pHluorin produces products with the same band sizes as IH01 and the gM-pHluorin plasmid. HSV-1. OK14 does not produce any detectable PCR product bands due to the lack of pHluorin sequence in the genome.
