# Supplementary material for: Individual herpes simplex virus 1 (HSV-1) particles exit by exocytosis and accumulate at preferential egress sites
Source: J Virol. 2024 Jan 9;98(2):e01785-23. doi: 10.1128/jvi.01785-23 (PMC10883806; doi:10.1128/jvi.01785-23)

Supplemental Figure 2

Western blot analysis to confirm deletion of gE, gI, and US9 in PRV IH01. PRV IH01 and PRV BaBe both show lack of bands for gE, gI, and US9, thus indicating the deletion of these genes in the parental strain and new recombinant.


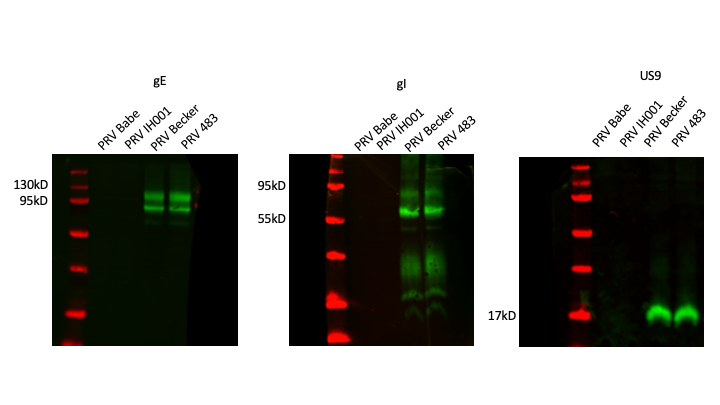

Supplement: Supplemental Material 2 — Supplemental figure showing western blot validation of a PRV recombinant virus. [file jvi.01785-23-s0002.docx]
